# SMAC-armed oncolytic virotherapy enhances the anticancer activity of PD1 blockade by modulating PANoptosis

**DOI:** 10.1186/s40364-025-00726-w

**Published:** 2025-01-09

**Authors:** Fanghui Chen, Liwei Lang, Jianqiang Yang, Fan Yang, Sijia Tang, Zhenzhen Fu, Nabil F. Saba, Ming Luo, Yong Teng

**Affiliations:** 1 Department of Hematology and Medical Oncology, Emory University, 201 Dowman Dr, Atlanta, GA 30322 USA; 2 Dental College of Georgia, Augusta University, Augusta, GA 30912 USA; 3 Winship Cancer Institute of Emory University, Atlanta, GA 30322 USA; 4 Department of Chemistry, Georgia State University, Atlanta, GA 30303 USA; 5 Wallace H. Coulter Department of Biomedical Engineering, Georgia Institute of Technology and Emory University, Atlanta, GA 30322 USA

**Keywords:** VSV-S, Head and neck cancer, PANoptosis, Immunotherapy, Antitumor immunity

## Abstract

**Background:**

Oncolytic viruses (OVs) are increasingly recognized as promising tools for cancer therapy, as they selectively infect and destroy tumor cells while leaving healthy cells unharmed. Despite considerable progress, the limited therapeutic efficacy of OV-based virotherapy continues to be a significant challenge in cancer treatment.

**Methods:**

The *SMAC*/*DIABLO* gene was inserted into the genome of vesicular stomatitis virus (VSV) to generate VSV-S. Head and neck squamous cell carcinoma (HNSCC) cell lines and orthotopic mouse models were employed for research. Morphological changes were observed using both light microscopy and transmission electron microscopy. Molecular alterations were analyzed through Western blotting and ELISA kits. The tumor secretome was characterized using a combination of biotinylation and LC-MS analysis. Immune cell changes were evaluated by flow cytometry and immunohistochemistry.

**Results:**

Compared to its parental virus, VSV-S not only increases apoptosis by overexpressing SMAC during VSV infection but also triggers elevated levels of PANoptosis (pyroptosis, apoptosis, and necroptosis) in HNSCC cells via activation of caspase-1/gasdermin D (GSDMD) signaling. As a result, VSV-S-induced PANoptosis promotes CD8^+^ T cell tumor infiltration and enhances their cytotoxic capacity, eventually potentiating T cell-mediated antitumor immunity. Moreover, VSV-S reduces PDL1 levels in HNSCC cells and, in combination with PD1 blockade, produces a more potent antitumor effect than either therapy alone.

**Conclusions:**

Our findings demonstrate that the combination of VSV-S and PD1 blockade offers a synergistic therapeutic strategy for HNSCC, supporting the advancement of VSV-based virotherapy as a promising strategy to improve outcomes for HNSCC patients.

**Supplementary Information:**

The online version contains supplementary material available at 10.1186/s40364-025-00726-w.

## Background

Conventional cancer treatments, such as chemotherapy and radiotherapy, often come with severe side effects and limited long-term efficacy, particularly for advanced and metastatic cases [1]. Even though immunotherapy has improved the prognosis for patients with recurrent or metastatic HNSCC, a large proportion of patients with this disease do not benefit from single agent PD1 inhibitors [1]. Oncolytic viruses (OVs) are gaining recognition as promising tools for cancer treatment as they selectively infect and destroy tumor cells while sparing healthy cells [2, 3]. Their mechanism of action involves the virus infecting and replicating in the cancer cells causing cell lysis, which releases infectious virus progeny to infect adjacent tumor cells. In addition, the lysis of cancer cells with OV infection may stimulate the host immune system to recognize and attack the tumor [3, 4]. One of the most well-studied OVs is vesicular stomatitis virus (VSV), which is a non-pathogenic RNA virus. VSV infection in humans is typically asymptomatic. Its broad tropism, rapid replication, and ease of genetic manipulation make it a valuable tool for cancer research [5]. In addition to the previously mentioned mechanisms, recent studies have demonstrated that VSV can induce endoplasmic reticulum (ER) stress-mediated apoptosis [6]. VSV has demonstrated efficacy in preclinical models of various cancers like ovarian cancer, breast cancer, pancreatic cancer, melanoma, and glioblastoma [7–10]. Several studies on VSV for cancer treatment have advanced to Phase 1/2 clinical trials [11, 12].

The limited efficacy of OVs in cancer treatment can be attributed to several factors, including immune system interference (the body’s immune system may recognize and eliminate OVs before it can effectively target and destroy cancer cells), the immunosuppressive tumor microenvironment (TME), heterogeneity of tumor cells, antiviral responses of tumor cells, and innate resistance to viral infections [2, 3]. Addressing these challenges may require the redesign of more effective OVs or the development of combination strategies in order to boost overall efficacy. In one study, VSV expressing murine IFN-β (VSV-IFN-β) demonstrated notable antitumor effects in lung cancer [4]. Another study combining the VSV-IFN-β with anti-PD1 antibody showed suppressed tumor growth and increased survival rate in a hepatocellular carcinoma mouse model. VSV-IFN-β treatment increased the generation of anti-tumor T cell populations, thereby significantly enhancing the efficacy of immune checkpoint inhibitors (ICIs) like anti-PD1 antibodies [4]. Strikingly, infection of HeLa cells with wild-type VSV (wtVSV) downregulated the second mitochondria-derived activator of caspases (SMAC), a protein released from mitochondria upon activation of the intrinsic apoptotic pathway [13], resulting in a reduced apoptotic rate in the infected cells [7]. The role of SMAC in activating apoptosis underscores its importance in maintaining the cell-killing efficacy of VSV in cancer cells. Therefore, we engineered VSV-S by inserting the *SMAC*/*DIABLO* gene into the VSV genome [7]. This modification enhances the induction of apoptosis in tumor cells, leading to significant tumor regression in breast cancer and pancreatic cancer models [8]. These studies suggest that oncolytic virotherapy has a great potential in treating various types of cancers with high efficiency.

In this study, we found that VSV-S exhibited significantly stronger antitumor activity in head and neck squamous cell carcinoma (HNSCC) cells compared to wtVSV. VSV-S not only enhances cancer cell apoptosis by overexpressing SMAC during VSV infection, but also induces PANoptosis (pyroptosis, apoptosis, and necroptosis), which promotes tumor trafficking of CD8^+^ T cells and enhances their cytotoxicity, thereby potentiating T cell-mediated antitumor immunity. In addition, we discovered that VSV-S suppresses PD-L1 levels in HNSCC cells and that combining PD1 inhibition with VSV-S results in a more potent antitumor effect than achieved with either treatment alone. Our findings provide preclinical data supporting the development of VSV-based antitumor therapies, which hold promise for improving treatment efficacy in patients with HNSCC.

## Methods

### Primary tissue specimens

Formalin-fixed paraffin-embedded (FFPE) tissues from HNSCC patients were obtained from the Head and Neck Satellite Tissue Bank of Emory University. All clinical specimens were obtained with written informed consent from the patients. The studies were conducted in accordance with recognized ethical guidelines (Declaration of Helsinki), and that the studies were approved by Emory Institutional Review Board.

### Cell lines and culture

Human HN12 cells were a kind gift from Dr. Andrew Yeudall (Augusta University, GA) in 2016 and maintained in our lab. Human CAL27 cells were purchased from American Type Culture Collection (ATCC). Mouse MOC2 cells were obtained from Kerafast (Boston, MA). All cells were used for experiments before passage 10 and cultured in complete DMEM medium (Thermo Fisher Scientific, Waltham, MA) containing 10% fetal bovine serum (FBS) (Biological Industries, Cromwell, CT, USA), 2 mM L-glutamine (Biological Industries, Cromwell, CT, USA) and 1% pen-strep (Sigma-Aldrich, St. Louis, MO, USA) at 37 °C in a humidified incubator supplied with 5% CO2. Luciferase stable HN12 cells (HN12-Luc) and MOC2 cells (MOC2-luc) were generated by transduction of pGL4.5 vector (Promega, Madison, WI, USA) and selection with hygromycin (Sigma-Aldrich, St. Louis, MO, USA) for six weeks. All cell lines were genetically authenticated and were routinely screened for mycoplasma contamination by MycoAlert Mycoplasma Detection Kit (Lonza).

### Reagents, antibodies, primers and constructs

MTS Assay Kit and D-luciferin bioluminescent substrate were purchased from Thermo Fisher Scientific (Waltham, MA, USA). Xevinapant (AT-406) and Z-DEVD-FMK were obtained from MCE (Monmouth Junction, NJ, USA). 2’,7’-dichlorofluorescein (DCF) and n-acetylcysteine (NAC) were purchased from Sigma-Aldrich (St. Louis, MO, USA). For gene knockdown, the pLKO.1-puro TRC control shRNA targeting the gene encoding green fluorescent protein (shGFP) and specific shRNAs targeting the human *GSDMD* gene (shGSDMD-1 and shGSDMD-2) were purchased from Horizon Discovery (Lafayette, CO, USA). ViraPower Lentiviral Packaging Mix contains an optimized mixture of the three packaging plasmids (pLP1, pLP2, and pLP/VSVG) was obtained from Invitrogen (Carlsbad, CA, USA). All antibodies and primers used in this study are listed in Supplementary Table S1 and Supplementary Table S2. Cell proliferation, viability, colony formation, quantitative reverse transcription polymerase chain reaction (qRT-PCR), and Western blot assays were carried out as previously described [14, 15]. Densitometric analysis of Western blots was performed using ImageJ Fiji (version 1.2; WS Rasband, National Institute of Health, Bethesda, MD, USA).

### Virus preparation and infection

VSV-S was engineered by inserting the *SMAC*/*DIABLO* gene into the VSV genome in the cDNA vector [7]. The recombinant viruses underwent two rounds of plaque purification. Virus stocks were propagated in HeLa cells and virus pellets were resuspended in PBS containing 5% sucrose and stored at -80 °C. For virus infection, cells were washed with Dulbecco’s PBS (DPBS), and the virus inoculum was added to cells at varying multiplicity of infections (MOIs). Virus uptake occurred at 37 °C for 1 h, followed by the addition of culture media without FBS, allowing the infection to proceed at 37 °C in 5% CO2 for the specified duration.

### Lentivirus-mediated gene knockdown

Lentiviral shRNA plasmids, along with packaging plasmids, were co-transfected into Lenti-Pac 293TA cells (GeneCopoeia, Rockville, MD, USA) using Lipofectamine 3000 (Invitrogen, Carlsbad, CA, USA) according to the manufacturer’s instructions. Forty-eight hours after transfection, virus particles were collected and used to infect the target cells to generate stable knockdown cell lines. The knockdown efficacy was then evaluated by Western blot.

### Enzyme-linked immunosorbent assays (ELISA)

LDH release was measured using the Pierce™ LDH Cytotoxicity Assay Kit (Thermo Fisher Scientific, Waltham, MA, USA) according to the manufacturer’s protocol. Briefly, culture medium was collected and centrifuged at 500 × g for 5 min to remove cell debris. LDH release into the medium was measured at OD 490. Relative LDH release was expressed as the percentage LDH activity in culture supernatants compared to total LDH (from media and cells) and used as an index of cytotoxicity. IL-1β levels in culture supernatants were measured with a Human IL-1 beta ELISA Kit (Abcam, Cambridge, MA, USA). HMGB1 levels in culture supernatants were measured with a Human HMGB1 ELISA kit (ArigoBio, Burlington, NC, USA).

### Transmission electron microscope (TEM)

TEM was performed according to our established protocol [15]. Briefly, 1.0 × 10^7^ HN12 or CAL27 cells were infected with wtVSV or VSV-S and their control cells were harvested and fixed in 2% glutaraldehyde for 45 min. The samples were postfixed in 2% osmium tetroxide for 2 h, dehydrated through a graded series of ethanol (50, 70, 90, and 100% for 15 min and then three times at 100%), and embedded in Epon-Araldite resin. Ultrathin sections were double stained with 1% lead citrate and 0.5% uranyl acetate and examined with a JEOL JEM-1230 TEM.

## Cell death and apoptosis

For in vitro analysis, apoptosis was determined by flow cytometry using the Annexin V: PE Apoptosis Detection Kit (Invitrogen, Carlsbad, CA, USA) with 7-AAD. Annexin V-positive cells were quantified and classified as apoptotic cells, while cells positive for both Annexin V and 7-AAD were quantified and classified as dead cells. For in vivo analysis, apoptosis was assessed by staining for DNA fragmentation using In Situ Cell Death Detection Kit, TMR red (Roche, South San Francisco, CA, USA). The average number of TUNEL-positive cells was counted in 20 random fluorescence microscope fields for each condition.

### CD8+ T cell migration assay

Human peripheral blood mononuclear cells (PBMCs) were collected from healthy donors at Emory University with Ficoll-Paque Premium density gradient centrifugation (Cytiva, Shrewsbury, MA, USA) as we previously described [16]. CD8^+^ T cells were isolated from the fresh PBMCs using an EasySep™ Human CD8 Positive Selection Kit II (Stemcell Technologies, Cambridge, MA, USA) according to the manufacturer’s instructions. For CD8^+^ T cell migration assay, 1 × 10^5^ HN12 cells were seeded in the lower chambers of transwells and infected with wtVSV or VSV-S. Simultaneously, 1 × 10^5^ CD8^+^ T cells were plated on the upper chamber, which contained a 5 μm porous polycarbonate membrane. CD8^+^ T cells that migrated into the lower chamber media were collected after 8 h and stained with CellTracker™ CM-Dil dye (Thermo Fisher Scientific, Waltham, MA, USA) for imaging. Migrated CD8^+^ T cells were counted from 10 random microscope fields for each condition.

### Generation of ER-BioID^HA^ stably expressing MOC2 cells (MOC2-ER)

ER-BioID^HA^ expression plasmid was a kind gift from Dr. Toren Finkel at University of Pittsburgh [17], which was designed to contain the BioID coding sequence with the following features in order: IgK signal peptide, BioID2 coding sequence, HA tag, and the ER retention signal KDEL (Lys-Asp-Glu-Leu) tetrapeptide. To express ER-BioID^HA^ in HNSCC cells, MOC2 cells were infected overnight with ER-BioID^HA^ lentivirus and infected cells were selected by 1.5 µg/ml puromycin for 2 weeks to generate the stable MOC2-ER cells.

### Liquid chromatography-mass spectrometry (LC-MS)

MOC2-ER cells were pulsed with 50 µM biotin for 12 h. Following two PBS washes to remove residual biotin from the media, the cells were infected with or without VSV-S for 24 h. The supernatant was collected, centrifuged at 200 g for 10 min, and carefully transferred to an Amicon^®^ Ultra Centrifugal Filter Tube with a 10 kDa molecular weight cutoff (MilliporeSigma, St. Louis, MI, USA). After centrifugation at 3000 g for 50 min at 4 °C, the conditioned medium was incubated with streptavidin beads (Invitrogen, Carlsbad, CA, USA) overnight. Beads were collected using a magnetic stand, washed at least five times with 50 mM ammonium bicarbonate, and resuspended for mass spectrometric detection at the Taplin Mass Spectrometry Facility at Harvard Medical School. LC-MS data were carefully analyzed using MaxQuant v2.5.2.0 software. The peak list was generated for extensive database searching. Different proteins (FC ≥ 2, *p* < 0.05) were compared. The resulting peaks were plotted on a two-dimensional graph with retention time on the x-axis and m/z ratio on the y-axis, providing a clear visual representation of the data.

### Flow cytometry

For characterization of tumor infiltrating lymphocytes, mucosal tumors excised from the indicated treatment groups were digested in X-Vivo-15 (Lonza, Durham, NC, USA) supplemented with Collagenase H (Sigma-Aldrich, St. Louis, MO, USA) and DNase (Roche, South San Francisco, CA, USA) and incubated at 37 °C, 5% CO2 for 30 min before being filtered through a 70 μm cell strainer. Single-cell suspensions from tumors were rinsed in FACS buffer, then stained with surface antibodies for 30 min, followed by additional rinsing and fixation. ArC™ Amine Reactive Compensation Bead Kit and LIVE/DEAD™ Fixable Aqua Dead Cell Stain Kit were purchased from Invitrogen (Carlsbad, CA, USA). The fluorochrome-conjugated antibodies used for flow cytometry are listed in Supplementary Table S1. Samples were analyzed on a BD Symphony A3 cytometer, then further analyzed using FlowJo (V.10.8.1) software (BD Biosciences, Franklin Lakes, NJ, USA). ‘Fluorescence minus one’ controls were tested for each multicolor flow panel.

### Animal studies and toxicity analysis

Six-week-old NOD.Cg-*Prkdcscid Il2rgtm1Wjl/SzJ* (NSG) and C57BL/6 mice were purchased from the Jackson Laboratory (Bar Harbor, ME, USA). All animal experiments were approved by the Institutional Animal Care and Use Committee (IACUC) of Emory University and Augusta University. To generate an orthotopic tongue tumor model for treatment assessment, 5 × 10^4^ HN12-luc cells or MOC2-luc cells were suspended in 50 µl of PBS/Matrigel (3:1) and injected into the anterior ~ 1/3 tongue of NSG and C57BL/6 mice, respectively [14, 18]. Seven days after cell inoculation, the mice were randomized to receive either MOCK (PBS) treatment or infection with wtVSV or VSV-S. ~3 × 10^6^ plaque-forming units (PFU) of wtVSV or VSV-S in 30 µL PBS with 5% sucrose were administered by intratumoral injection every three days, for a total of two doses. Two weeks after treatment, mice were intraperitoneally injected with D-luciferin bioluminescent substrate (Sigma-Aldrich, St. Louis, MO, USA), and tumor growth and metastasis were assessed by bioluminescence imaging (BLI) using a Xenogen IVIS-200 In Vivo Imaging System (PerkinElmer, Waltham, MA, USA). Tumor dimensions were measured with electronic calipers, and tumor volume was calculated by the formula of V = length × width^2^ × 1/2. To generate an orthotopic buccal mucosal tumor model to evaluate the treatment efficacy of the combination of VSV-S and αPDL1, 1 × 10^6^ MOC2 cells were implanted into C57BL/6 mice by intramucosal injection [16, 18, 19]. On day 7 after cell inoculation, mice were randomized to receive MOCK treatment (Rat IgG2a isotype control, Bio X Cell, Lebanon, NH, USA), or treatment with VSV-S and/or αPD1. A dose of 3 × 10^6^ PFU of VSV-S was administered via intratumoral injection every three days for a total of two doses. InVivoMAb anti-mouse PD1 antibody (Clone: 29 F.1A12, Bio X Cell, Lebanon, NH, USA) was administered intraperitoneally at a dose of 200 µg per injection every three days for a total of three doses, starting one day after the first dose of VSV-S. Tumor dimensions were measured twice a week with electronic calipers, and tumor volume was calculated by the formula of V = length × width^2^ × 1/2. The body weight and physical activity of each animal were followed as markers of toxicity. When the animal experiment was terminated, mice were sacrificed, and blood was collected via the ocular vein for the determination of serum levels of alanine transaminase (ALT), aspartate transaminase (AST), and creatinine. ALT and AST were measured using the EnzyChrom™ Alanine Transaminase Assay Kit and Aspartate Transaminase Assay Kit (BioAssay Systems, Hayward, CA, USA), respectively. Serum creatinine levels were measured using the Creatinine Assay Kit (Cayman Chemical, Ann Arbor, MI, USA). Tumors were excised for flow cytometry and immunohistochemistry (IHC) analysis.

### IHC

Tissue sections were deparaffinized with xylene, rehydrated through a graded alcohol series, and incubated in 3% hydrogen peroxide. Sections were placed in 10 mM sodium citrate buffer (pH 6.0) at sub-boiling temperatures for 10 min and incubated with 10% normal goat serum, followed by incubation with the indicated primary antibodies. Immunoreactivity was visualized using the DAB Detection kit (Vector laboratories, Burlingame, CA, USA), and slides were counterstained with hematoxylin, dehydrated, mounted, and scanned using the Olympus Nanozoomer whole-slide scanner. The German semi-quantitative scoring method was used to examine the final immunoreactivity score for SMAC in patient tissue specimens as we described previously [16, 20]. Briefly, each specimen was scored for intensity (no staining = 0; weak staining = 1; moderate staining = 2; strong staining = 3) and for extent of stained cells (0% = 0; 1–24% = 1; 25–49% = 2; 50–74% = 3; 75–100% = 4). Signal Index (SI) was the product of the intensity score multiplied by the extent score. To evaluate the levels of c-caspase 1, IL1β, p-MLKL in mouse tumor tissue, IHC staining was quantified using Image pro-Plus6.0 software (Media Cybernetics, Silver Springs, MD, USA) and presented as integrated optical density (IOD). The quantitation of positive CD8 cells in the tumor sections was counted in 10 random fields and the average number of positive cells per reported field was calculated based on the results provided by three investigators who were blind to pathological information.

### Bioinformatics and statistical analysis

The mRNA expression data from the Cancer Genome Atlas (TCGA) HNSCC cohort was obtained from the GDC Data Portal (https://portal.gdc.cancer.gov/projects/TCGA-HNSC). To determine the impact of *BIABLO* expression on overall survival of HNSCC patients, Kaplan-Meier survival analysis was performed based on the patient groups stratified by the high and low gene expression in TCGA HNSCC dataset. Data were analyzed using statistical software GraphPad Prism 9 (San Diego, CA, USA). Experimental values are expressed as mean ± standard deviation (SD). For comparison between two groups, statistical analysis was performed using unpaired, two-tailed Student’s t-test. One-way analysis of variance (ANOVA) followed by Tukey’s multiple testing correction was used for comparison of more than two groups. *p* values less than 0.05 were considered statistically significant.

## Results

### The combination of the SMAC mimetic and wtVSV leads to enhanced HNSCC cell apoptosis

**Fig. 1:**
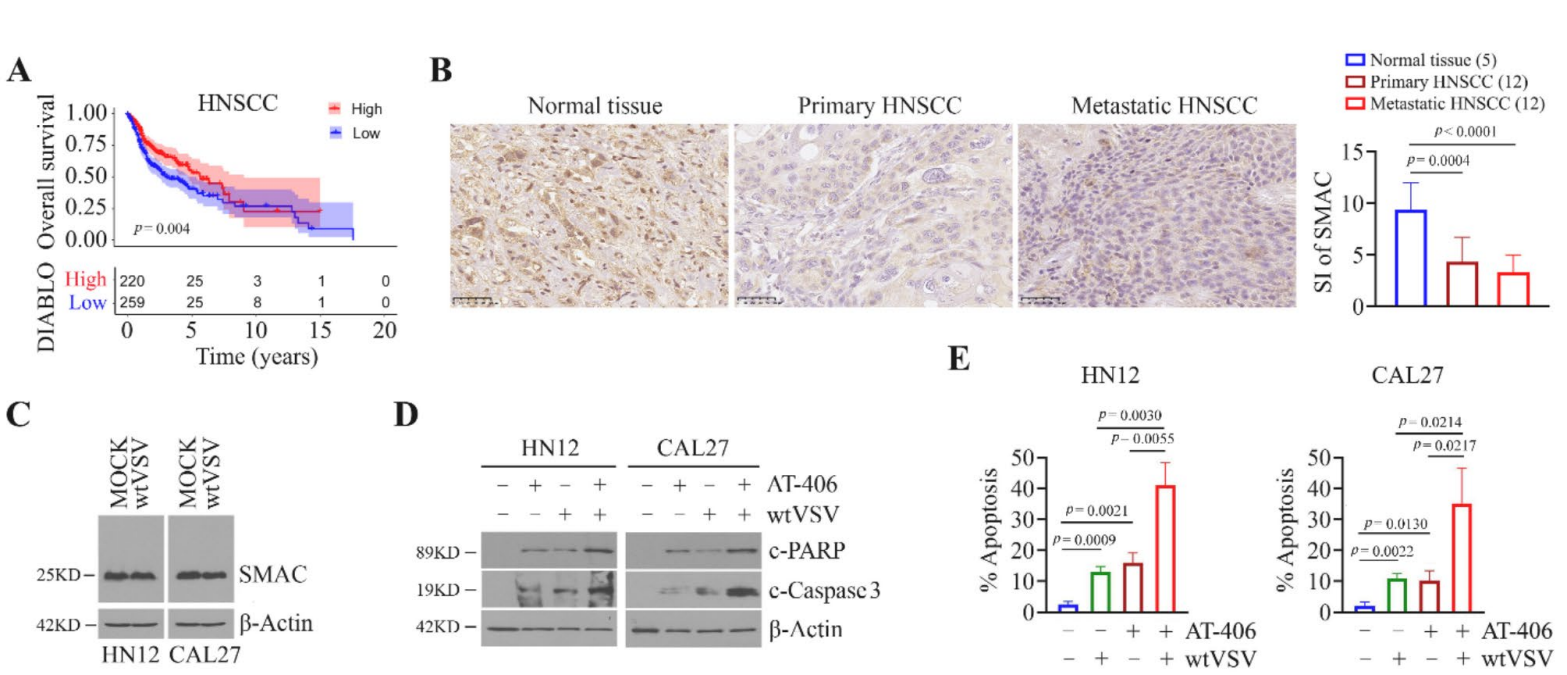
Addition of the SMAC mimetic AT-406 to wtVSV leads to enhanced apoptosis in HNSCC cells. (**A**) Overall survival analysis for HNSCC patients based on *DIABLO* gene expression (high vs. low) in TCGA HNSCC cohort. (**B**) IHC for SMAC in primary and metastatic human HNSCC tissues and normal counterparts. Representative IHC images and quantitative signal index (SI) of SMAC immunostaining are shown in the left and right panels, respectively. Statistical significance was evaluated using one-way ANOVA with Tukey’s adjustments for multiple comparisons. (**C**) Effect of wtVSV infection on SMAC expression in HN12 and CAL27 cells. (**D**) Effect of wtVSV infection alone or in combination with the SMAC mimetic AT-406 on the cleavage of PARP and caspase 3 in HN12 and CAL27 cells determined by Western blot. (**E**) Apoptosis of HN12 and CAL27 cells treated with wtVSV or AT-406 or their combination determined by flow cytometry with 7-AAD and Annexin V double staining. Quantitative data (*n* = 3) of flow cytometry are shown. Statistical significance was determined by unpaired, two-tailed Student’s t test. In (**D**) and (**E**), HN12 and CAL27 cells were infected with wtVSV (MOI = 5) and/or 5 µM AT-406 for 24 h before apoptosis measurement

To explore the clinical relevance of *SMAC*/*DIABLO* gene expression, we conducted a bioinformatics analysis using the Cancer Genome Atlas (TCGA) HNSCC database. Survival analysis revealed a strong correlation between high SMAC/DIABLO expression and increased overall survival in HNSCC patients (Fig. 1A). Since HPV-negative (HPV-) HNSCC carries a less favorable prognosis than HPV-positive (HPV+) disease [21, 22], we focused more on HPV- subtype. IHC analysis of tumor tissues from untreated HPV- HNSCC patients further showed that SMAC protein levels were much lower in primary HNSCC tissue compared with the normal counterparts (Fig. 1B). No significant difference was seen in SMAC immunostaining between primary and metastatic HNSCC samples (Fig. 1B).

Endogenous mitochondrial SMAC (25KD), the mitochondrial targeting signal of 55 residues cleaved from the N-terminus of SMAC precursor (27KD), was detected in both HNSCC HN12 and CAL27 cell lines (Fig. 1C). We then determined SMAC level changes in these two cell lines following VSV infection. After 4 h of incubation with wtVSV at a MOI of 5, nearly 100% of HN12 cells were successfully infected (Supplementary Fig. S1). However, this highly efficient infection did not alter SMAC expression in HN12 and CAL27 cells (Fig. 1C and Supplementary Fig. S2). This differed from observations in wtVSV-infected HeLa cells, where SMAC expression was downregulated compared to MOCK-infected cells [13], suggesting that this effect may be cell context-dependent and vary based on the cellular environment. AT-406 (Xevinapant) is a potent and orally bioavailable SMAC mimetic, inducing rapid degradation of cellular cIAP1 protein [23]. Similar to treatment with wtVSV, AT-406 significantly enhanced HNSCC cell apoptosis, which was accompanied by the appearance of PARP and caspase 3 cleavage (Fig. 1D and E). More strikingly, the combination of AT-406 and wtVSV induced a greater number of apoptotic cells than either treatment alone, as evidenced by a higher apoptotic rate and increased levels of cleaved PARP and caspase 3 (Supplementary Fig. S3, Fig. 1D and E). These findings suggest that increasing *SMAC*/*DIABLO* expression in HNSCC cells could potentially enhance the antitumor efficacy of wtVSV.

### VSV-S exhibits more potent antitumor activity compared to wtVSV

**Fig. 2:**
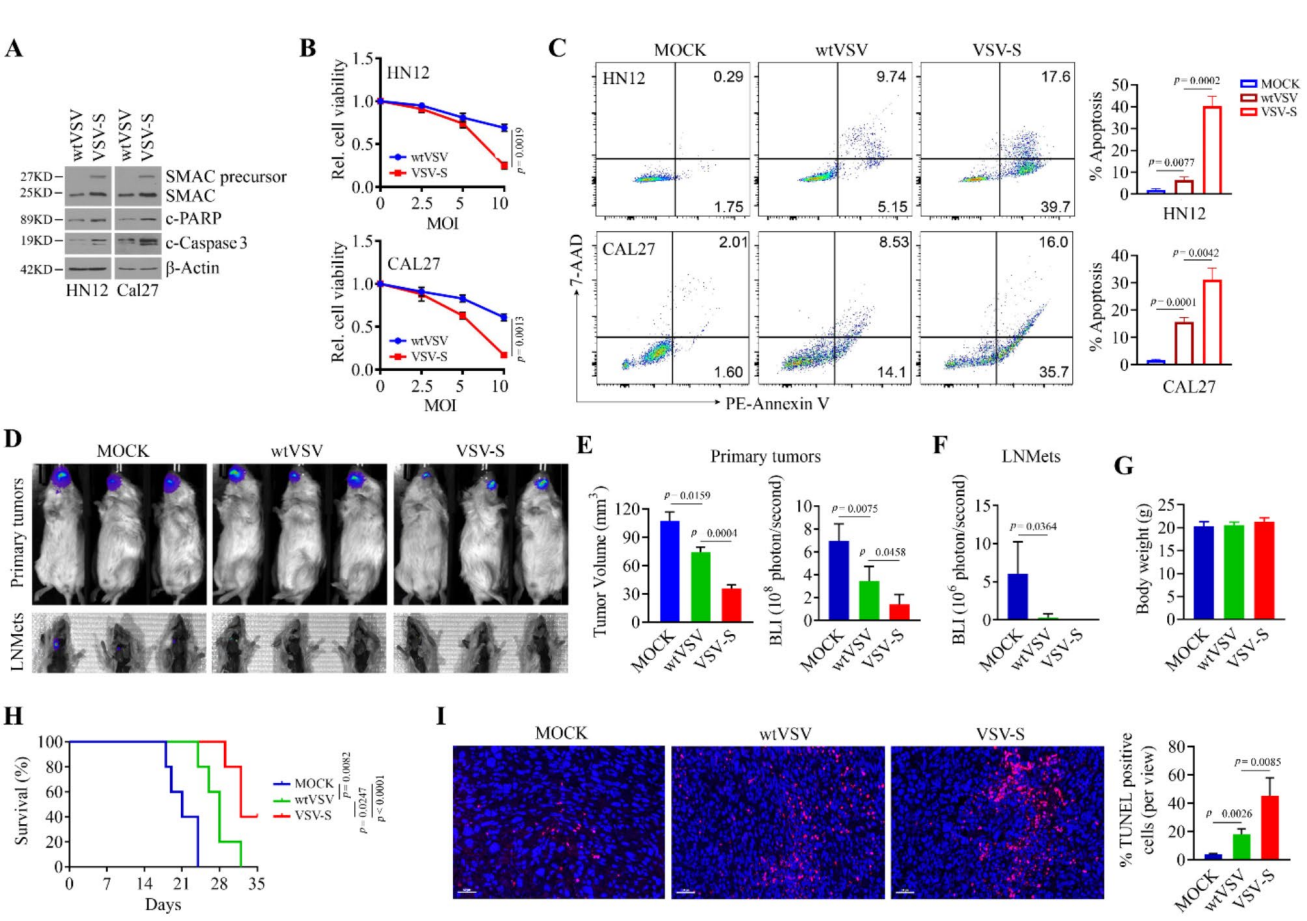
VSV-S exhibits more potent anti-HNSCC activity compared to wtVSV. (**A**) Effect of wtVSV and VSV-S infection on SMAC expression and cleavage of PARP and caspase 3 in HN12 and CAL27 cells. (**B**) Effect of wtVSV and VSV-S infection on the viability of HN12 and CAL27 cells. (**C**) Apoptosis and total cell death of HN12 and CAL27 cells infected with wtVSV or VSV-S determined by flow cytometry after 7-AAD and Annexin V double staining. Representative flow images and quantitative data (*n* = 3) are shown in the left and right panels, respectively. (**D**) Representative bioluminescence images of tongue tumors and tumor metastasis in lymph nodes in HN12 tumor-bearing NSG mice captured three weeks after cell inoculation. (**E**) Quantitative data of tumor volume (left) and bioluminescence intensity of tongue tumors (right) in three treatment groups (*n* = 5 mice/group). (**F**) Quantitative bioluminescence intensity of lymph node metastatic tumors in three treatment groups (*n* = 5 mice/group). (**G**) Effect of wtVSV or VSV-S infection on body weight of treated mice (*n* = 5 mice/group). In (**E**-**G**), statistical significance was evaluated using one-way ANOVA with Tukey’s adjustments for multiple comparisons. (H) Survival curve of HN12 tumor-bearing mice infected with wtVSV or VSV-S (*n* = 5 mice/group). Survival curves were compared using the Log-rank (Mantel-Cox) test for multiple groups. (**I**) TUNEL analysis in HN12 tumor tissues isolated from NSG mice infected with wtVSV or VSV-S (*n* = 5 mice/group). Statistical significance was determined by unpaired, two-tailed Student’s t test

We constructed VSV-S, a next-generation oncolytic VSV, by inserting the *SMAC*/*DIABLO* gene into the genome of VSV [7]. VSV-S significantly elevated SMAC precursor expression in HN12 and CAL27 cells, leading to an increase in mitochondrial SMAC compared to wtVSV infection (Fig. 2A and Supplementary Fig. S2). VSV-S infection exhibited more potent cytotoxic activity than the infection of the parental virus in both HN12 and CAL27 cells, as indicated by increased levels of cleaved PARP and caspase 3, reduced cell viability and higher apoptosis (Fig. 2A and C). Consistently, VSV-S induced HN12 tumor remission more effectively than wtVSV in NSG mice with the orthotopic tongue tumors (Fig. 2D and E). After four weeks of cell inoculation, cervical lymph node metastases (LNMets) were observed in HN12 tumor-bearing mice with mock treatment, as indicated by the bioluminescence signal in lymph nodes (Fig. 2D and F). The bioluminescence intensity in the lymph nodes was significantly lower in mice treated with wtVSV compared to those with mock treatment (Fig. 2D and F). Notably, VSV-S infection completely prevented the development of metastatic tumors in lymph nodes of HN12 tumor-bearing mice (Fig. 2D and F). Neither wtVSV nor VSV-S treatment affected the body weight of mice (Fig. 2G), indicating no significant systemic toxicity associated with these treatments. Additionally, tumor-bearing mice treated with VSV-S had a longer survival compared to those receiving mock or wtVSV treatment (Fig. 2H). IHC further revealed a higher number of TUNEL-positive cells in HN12 tumors treated with VSV-S than in those treated with mock or wtVSV (Fig. 2I). These findings suggest that VSV-S demonstrates antitumor efficacy at least in part by enhancing apoptosis in HNSCC cells.

### VSV-S triggers PANoptosis in HNSCC cells with greater effectiveness than wtVSV

**Fig. 3:**
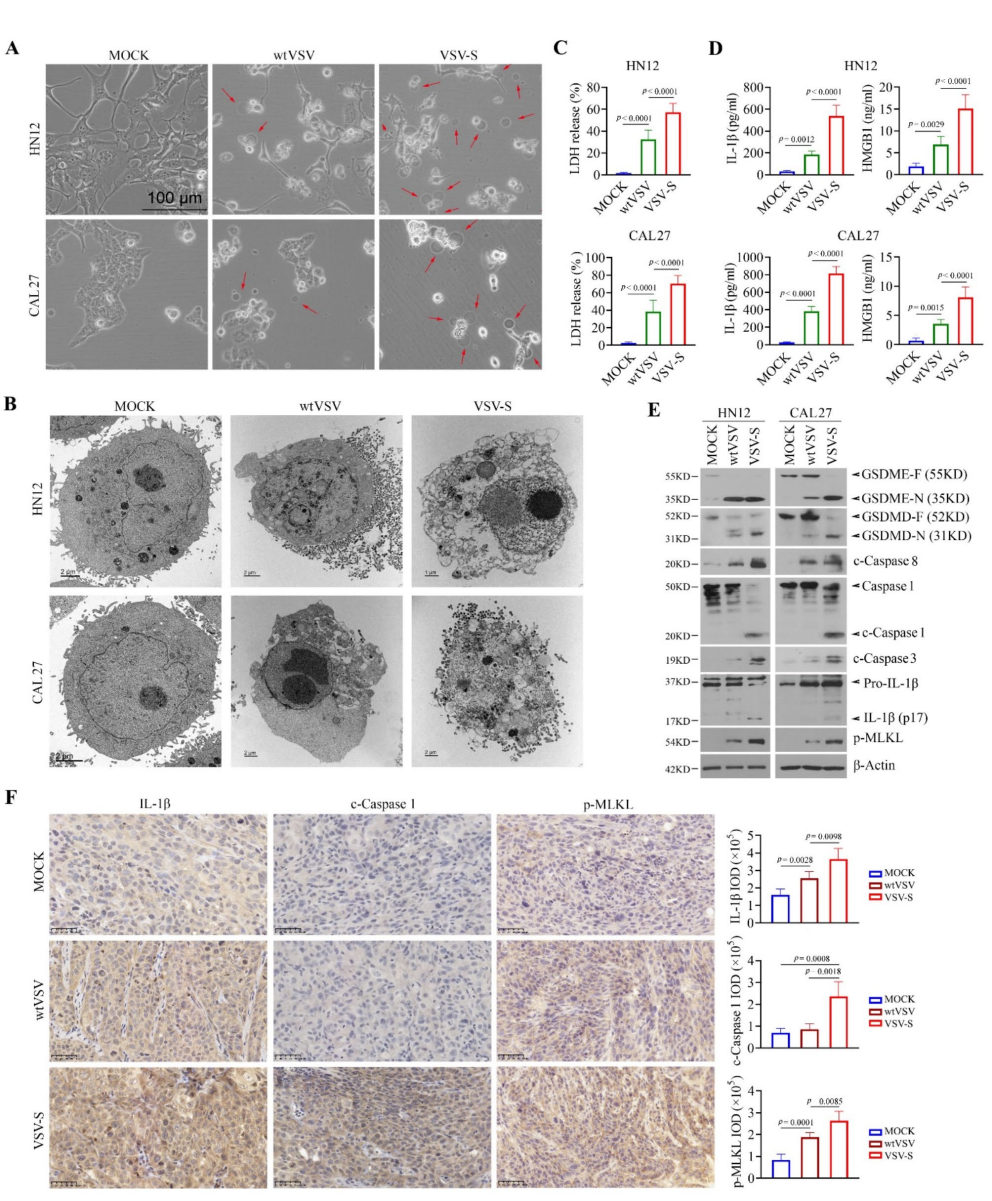
VSV-S induces PANoptosis in HNSCC cells more effectively than wtVSV. (**A**) Morphology of HN12 and CAL27 cells infected with wtVSV or VSV-S. Red arrows indicate pyroptotic cells. (**B**) TEM images of HN12 and CAL27 cells infected with wtVSV or VSV-S. (**C**) LDH release in HN12 and CAL27 cells infected with wtVSV or VSV-S determined by the Pierce™ LDH Cytotoxicity Assay Kit (*n* = 6 repeats). Statistical significance was evaluated using one-way ANOVA with Tukey’s adjustments for multiple comparisons. (**D**) Levels of IL-1β and HMGB1 in the supernatant of HN12 and CAL27 cells infected with wtVSV or VSV-S determined by ELISA (*n* = 6 repeats). Statistical significance was evaluated using one-way ANOVA with Tukey’s adjustments for multiple comparisons. (**E**) PANoptotic signaling in wtVSV- or VSV-S-infected HN12 and CAL27 cells determined by Western blot with multiple PANoptosis markers. (**F**) IHC for IL-1β, c-caspase 1 and p-MLKL in HN12 tumor tissues isolated from NSG mice infected with wt-VSV or VSV-S. Representative IHC images and quantitative data (*n* = 10 random field/slide/group) are shown in the left and right panels, respectively. Statistical significance was determined by unpaired, two-tailed Student’s t test

Pyroptosis is a major form of inflammatory cell death triggered by viral infections [24, 25]. Interestingly, the formation of cell membrane pores and membrane rupture, well-defined morphological characteristics, were observed in HN12 and CAL27 cells when infected with wtVSV or VSV-S (Fig. 3A). VSV-S induces HN12 and CAL27 cell pyroptosis more efficiently than wtVSV, as demonstrated by a greater number of cell membrane pores along with increased lactate dehydrogenase (LDH) release and elevated levels of IL-1β and HMGB1 in the supernatant of infected cells (Fig. 3A and D). At the molecular level, either wtVSV or VSV-S infection led to the appearance of cleaved caspase 3, caspase 8, and N-terminal gasdermin E (GSDME-N) (Fig. 3E), supporting the notion that VSV can trigger caspase 3/8/GSDME-dependent pyroptosis regardless of *SMAC/DIABIO* insertion. Moreover, the appearance of cleaved caspase-1 and elevated levels of N-terminal GSDMD (GSDMD-N) and IL-1β were observed in VSV-S-infected HN12 and CAL27 cells compared to those cells infected with wtVSV (Fig. 3E), suggesting that VSV-S may enhance VSV-mediated pyroptosis by activating caspase 1-mediated GSDMD cleavage. We also assessed changes in the phosphorylation levels of mixed lineage kinase domain-like protein (MLKL), a necroptosis biomarker, and observed increased p-MLKL levels in cells infected with either wtVSV or VSV-S, with a more pronounced increase in cells infected with VSV-S (Fig. 3E). This suggests that the addition of SMAC to VSV enhances PANoptosis in HNSCC cells. Consistently, IHC analysis of HN12 tumor tissues isolated from the NSG xenograft mouse models subjected to mock, wtVSV or VSV-S infection (Fig. 2) revealed significantly elevated levels of IL-1β and p-MLKL in both wtVSV- and VSV-S-infected groups (Fig. 3F). In line with the in vitro findings, increased cleaved caspase-1 was only observed in tumors infected with VSV-S, compared to the other two treatment groups (Fig. 3F).

### ROS-associated apoptosis enhances caspase 1/GSDMD dependent pyroptosis during VSV-S infection

**Fig. 4:**
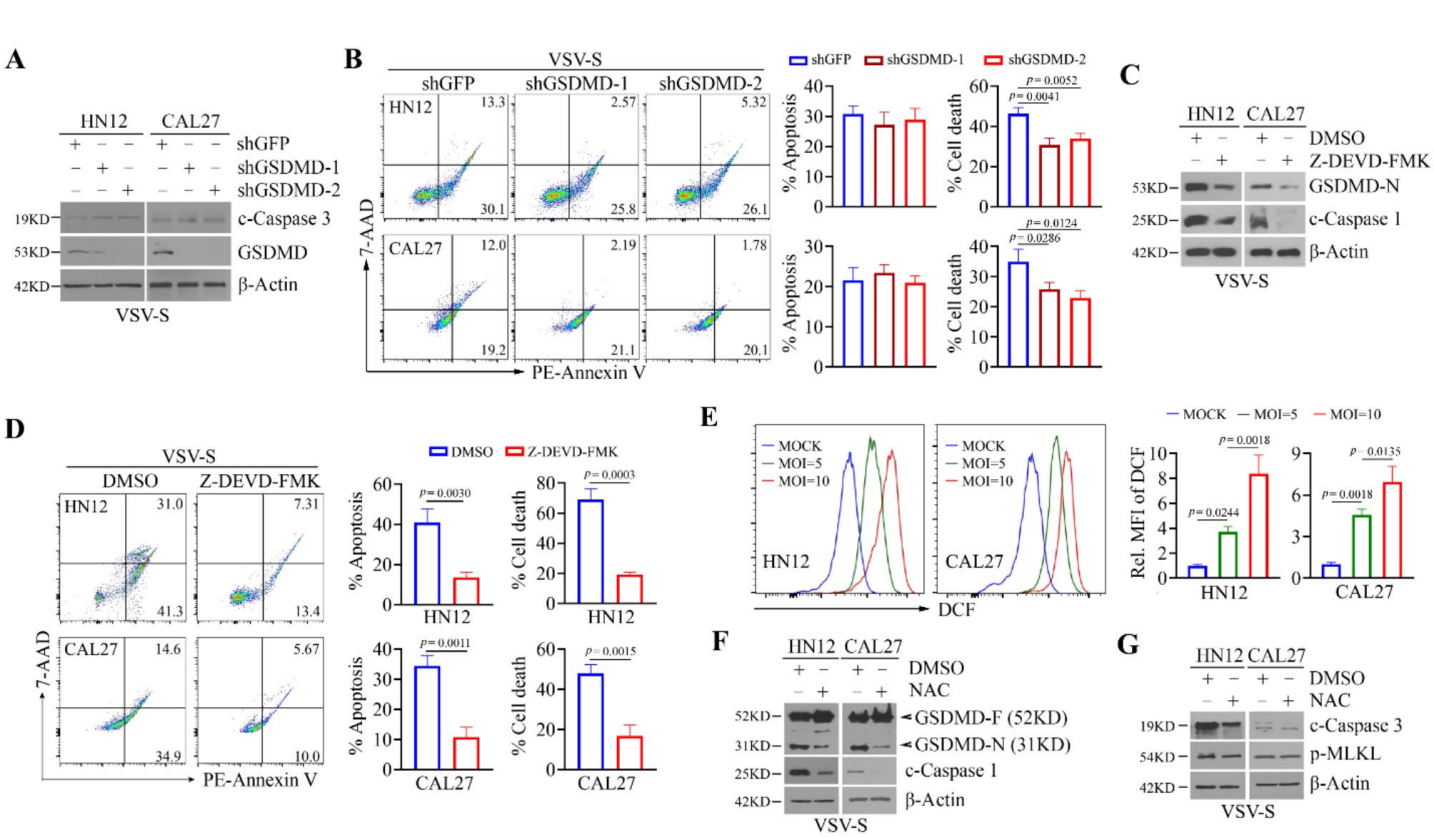
ROS-associated apoptosis enhances caspase 1/GSDMD dependent pyroptosis in HNSCC cells infected with VSV-S. (**A**) Effect of GSDMD knockdown on the cleavage of caspase 3 in VSV-S-infected HN12 and CAL27 cells determined by Western blot. (**B**) Effect of GSDMD knockdown on apoptosis and total cell death in VSV-S-infected HN12 and CAL27 cells determined by flow cytometry after 7-AAD and Annexin V double staining. Representative flow images and quantitative data (*n* = 3 repeats) are shown in the left and right panels, respectively. Statistical significance was determined by unpaired, two-tailed Student’s t test. (**C**) Effect of Z-DEVD-FMK treatment on the cleavage of GSDMD and caspase 1 in VSV-S-infected HN12 and CAL27 cells determined by Western blot. (**D**) Effect of Z-DEVD-FMK treatment on apoptosis and total cell death in VSV-S-infected HN12 and CAL27 cells determined by flow cytometry after 7-AAD and Annexin V double staining. Representative flow images and quantitative data (*n* = 3 repeats) are shown in the left and right panels, respectively. Statistical significance was determined by unpaired, two-tailed Student’s t test. (**E**) Intracellular ROS levels in VSV-S-infected HN12 and CAL27 cells determined by flow cytometry after DCF staining. Representative flow images and quantitative data (*n* = 3 repeats) are shown in the left and right panels, respectively. Statistical significance was evaluated using one-way ANOVA with Tukey’s adjustments for multiple comparisons. (**F**) Effect of NAC treatment on cleavage of caspase 1 and GSDMD in VSV-S-infected HN12 and CAL27 cells determined by Western blot. (**G**) Effect of NAC treatment on cleavage of caspase 3 and phosphorylation of MLKL in VSV-S-infected HN12 and CAL27 cells determined by Western blot. In (**F**) and (**G**), HN12 and CAL27 cells were infected with VSV-S (MOI = 5), in the presence or absence of 10 mM NAC for 24 h

To investigate the role of GSDMD in VSV-S-mediated antitumor effects, we specifically depleted GSDMD using lentiviral shRNAs (Fig. 4A and Supplementary Fig. S2). Knockdown of GSDMD reduced total cell death in HN12 and CAL27 cells infected with VSV-S compared to the knockdown control (Fig. 4B), without affecting c-caspase 3 levels and apoptotic rate (Fig. 4A and B), suggesting that GSDMD-dependent pyroptosis does not impact VSV-S-mediated apoptosis. Treatment with caspase 3 inhibitor, Z-DEVD-FMK, not only attenuated VSV-S-induced apoptosis in HN12 and CAL27 cells, but also reduced protein levels of c-caspase 1 and GSDMD-N, leading to a significant decrease in total cell death (Fig. 4C and D and Supplementary Fig. S2). We also examined changes in the levels of cellular reactive oxygen species (ROS) in HNSCC cells following VSV-S infection. DCF staining showed that VSV-S induced an increase in ROS in both HN12 and CAL27 cells and this effect was dose-dependent (Fig. 4E). Moreover, treatment with the ROS scavenger NAC prior to VSV-S infection led to decreased levels of cleaved caspase-1 and GSDMD-N in HN12 and CAL27 cells (Fig. 4F and Supplementary Fig. S2). NAC also attenuated c-caspase 3 levels in these cells when infected with VSV-S, but it did not affect p-MLKL levels (Fig. 4G and Supplementary Fig. S2). These findings indicate that ROS-associated apoptosis enhances caspase 1/GSDMD dependent pyroptosis during VSV-S infection.

### VSV-S promotes CD8+ T cell infiltration into head and neck tumors more effectively than wtVSV

**Fig. 5:**
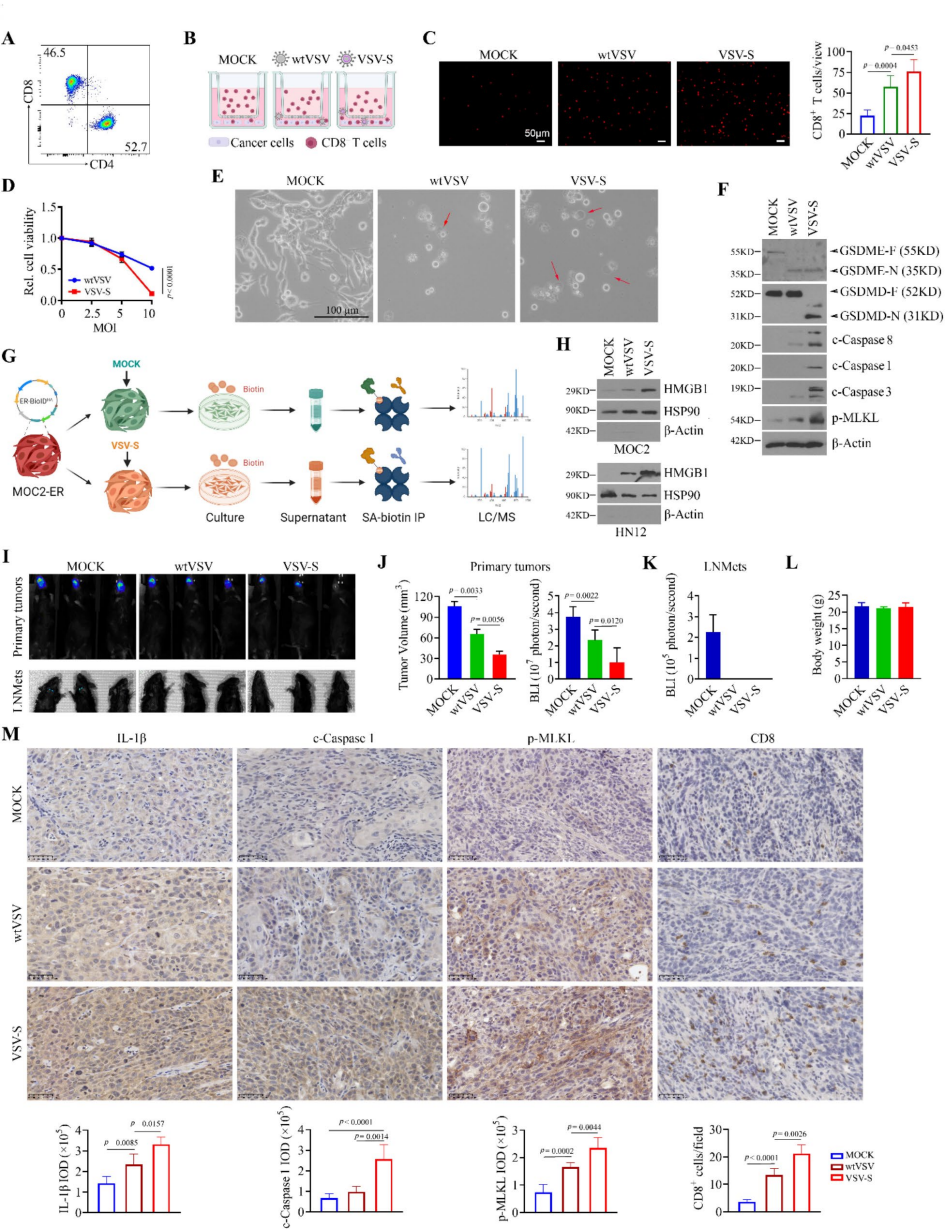
VSV-S significantly enhances CD8^+^ T cell infiltration into head and neck tumors compared to wtVSV. (**A**) Percent of CD8^+^ T cell subsets in monocytes isolated from human PBMCs determined by flow cytometry. (**B**) Schematic diagram of in vitro CD8^+^ T cell migration assay in the treatment of wtVSV or VSV-S. (**C**) Effect of wtVSV and VSV-S infection on CD8^+^ T cell migration to HN12 cells. Representative images with CellTracker™ CM-Dil dye and quantitative data (*n* = 10 field/group) are shown in the left and right panels, respectively. Statistical significance was evaluated using one-way ANOVA with Tukey’s adjustments for multiple comparisons. (**D**) Effect of wtVSV and VSV-S infection on MOC2 cell viability (*n* = 6 repeats). Statistical significance was determined by unpaired, two-tailed Student’s t test. (**E**) Morphology of MOC2 cells infected with wtVSV or VSV-S. Red arrows indicate pyroptotic cells. (**F**) PANoptotic signaling in wtVSV- or VSV-S-infected MOC2 cells determined by Western blot with PANoptosis markers. (**G**) Schematic of the process of assessing differential secretome of MOC2-ER cells infected with MOCK or VSV-S. MOC2-ER cells stably expressing ER-BioID^HA^ were infected with MOCK or VSV-S (MOI = 5) for 24 h, and supernatant was collected and incubated with SA beads, followed by LC-MS analysis. (**H**) HMGB1 levels in the supernatants of MOC2 or HN12 cells infected with wtVSV or VSV-S determined by Western blot. (**I**) Representative bioluminescence images of tongue tumors and tumor metastasis in lymph nodes in MOC2 tumor-bearing C57BL/6 mice captured three weeks after cell inoculation. (**J**) Quantitative data of tumor volume (left) and bioluminescence intensity of tongue tumors (right) in three treatment groups (*n* = 5 mice/group). Statistical significance was determined by unpaired, two-tailed Student’s t test. (**K**) Quantitative bioluminescence intensity of lymph node metastatic tumors in three treatment groups (*n* = 5 mice/group). (**L**) Effect of wtVSV or VSV-S infection on body weight of treated mice (*n* = 5 mice/group). (**M**) IHC for PANoptosis markers (IL-1β, c-caspase 1 and p-MLKL) and CD8 in MOC2 tumor tissues isolated from C57BL/6 mice infected with wt-VSV or VSV-S. Representative IHC images and quantitative data (*n* = 10 random field/slide/group) are shown. Statistical significance was determined by unpaired, two-tailed Student’s t test

Recent evidence indicates that PANoptosis induction in tumor cells leads to a robust inflammatory response and marked tumor regression [26]. Next, we sought to establish the link between VSV-S and antitumor immune response. In vitro T cell tumor migration assay was performed using the CD8^+^ T cells isolated from human PBMCs (Fig. 5A). In this assay, isolated human CD8^+^ T cells were seeded into the insert of a transwell, and their migration ability to HN12 cells was determined in the presence or absence of wtVSV or VSV-S (Fig. 5B). A significantly higher number of CD8 + T cells migrated to HN12 cells infected with either wtVSV or VSV-S compared to MOCK infection, with an even greater number observed in cells infected with VSV-S compared to wtVSV (Fig. 5C). CXCL9 and CXCL10 are key chemokines that facilitate T cell infiltration into tumors by recruiting effector T cells to the TME. In line with the migration data, VSV-S-infected HNSCC cells exhibited elevated expression and secretion levels of CXCL9 and CXCL10 compared to MOCK- or wtVSV-infected cells (Supplementary Fig. S4), suggesting that VSV-S promotes T cell trafficking to tumors not only through releasing PANoptosis-associated pathogen-associated molecular patterns (PAMPs) and/or damage-associated molecular patterns (DAMPs) into the TME, but also by enhancing the production of T cell-attracting chemokines.

Next, we studied the role of VSV-S in antitumor immunity using mouse HNSCC MOC2 cells. VSV-S induced PANoptosis-associated cell death more efficiently than wtVSV in MOC2 cells, as evidenced by cell viability, and morphological and molecular changes (Fig. 5D and F). To capture the tumor-specific secretome while excluding the influence of serum proteins, we transfected an ER lumen-resident BioID expression plasmid (ER-BioID^HA^) into MOC2 cells and generated MOC2-ER (Fig. 5G). These cells were then infected with or without VSV-S in the presence of biotin. After 24 h, the cell supernatants were collected, pulled down using streptavidin (SA), and subsequently analyzed by liquid chromatography-mass spectrometry (LC-MS) (Fig. 5G). This study identified 189 differentially expressed proteins (DEPs) that were exclusively present in the supernatants of VSV-S-infected MOC2-ER cells compared to control cells. Among them, HMGB1 was one of the proteins with the highest sequence coverage based on LC-MS analysis (Supplementary Fig. S5 and Fig. S6). Western blot analysis confirmed enhanced HMGB1 secretion in both VSV-S-infected MOC2 and HN12 cells, with levels significantly higher than those in cells infected with wtVSV (Fig. 5H). These findings further demonstrate that VSV-S has a greater potential to induce pyroptosis in HNSCC than wtVSV. In the syngeneic orthotopic tumor mouse models, either wtVSV or VSV-S infection remarkably reduced the primary tumor burden and the metastatic potential to cervical lymph nodes, without affecting body weight of MOC2 tumor-bearing mice (Fig. 5I-L). Consistent with the results from NSG mice, VSV-S demonstrated a stronger antitumor effect than wtVSV in immunocompetent C57BL/6 mice (Fig. 5I and K). Significantly higher levels of PANoptosis biomarkers, including IL-1β, cleaved caspase 1, and p-MLKL, were detected in VSV-S-infected MOC2 tumors compared to those infected with wtVSV(Fig. 5M). Additionally, IHC analysis revealed that VSV-S infection led to greater infiltration of CD8^+^ T cells in MOC2 tumors (Fig. 5M), suggesting that VSV-S may promote cytotoxic T cell infiltration through enhanced PANoptosis.

### VSV-S is more effective than wtVSV in enhancing the cytotoxic activity of CD8+ T cells

**Fig. 6:**
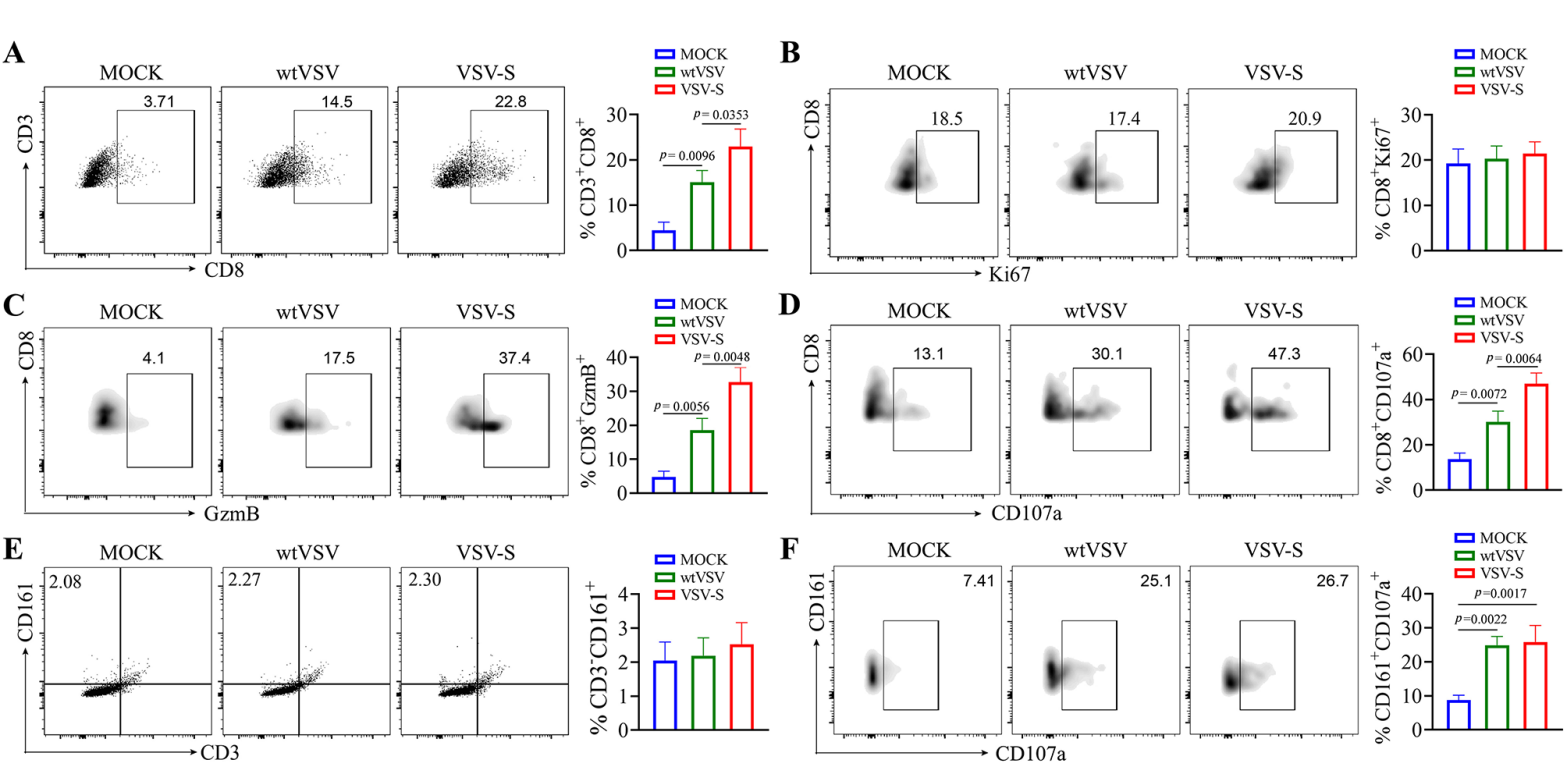
VSV-S enhances the cytotoxic activity of CD8^+^ T cells more effectively than wtVSV. (**A**) Percent of CD8^+^ T cells in MOC2 tumors infected with wtVSV or VSV-S determined by flow cytometry. (**B**) Percent of proliferative (Ki67^+^) CD8^+^ T cell subset in MOC2 tumors infected with wtVSV or VSV-S determined by flow cytometry. (**C**, **D**) Percent of cytotoxic (GzmB^+^ or CD107a^+^) CD8^+^ T cell subset in MOC2 tumors infected with wtVSV or VSV-S determined by flow cytometry. (**E**) Percent of NK cells in MOC2 tumors infected with wtVSV or VSV-S determined by flow cytometry. (**F**) Percent of cytotoxic (CD107a^+^) NK cell subset in MOC2 tumors infected with wtVSV or VSV-S determined by flow cytometry (*n* = 5 mice/group). Statistical significance was evaluated using one-way ANOVA with Tukey’s adjustments for multiple comparisons

To further understand the role of VSV-S in immunomodulation, we analyzed the proliferation and cytotoxic potential of tumor-infiltrating CD8^+^ T cells in MOC2 tumors infected with wtVSV or VSV-S. Consistent with the results from IHC (Fig. 5M), VSV-S infection increased CD8^+^ cells in MOC2 tumors compared to wtVSV infection (Fig. 6A). Moreover, increased populations of cytotoxic (GzmB^+^ or CD107a^+^) CD8^+^ T cells, but not proliferative (Ki67^+^) CD8^+^ T cells, were observed in MOC2 tumors infected with VSV-S compared to those infected with wtVSV (Fig. 6B and D). Interestingly, the number of tumor-infiltrating NK cells in MOC2 tumors remained unchanged with or without viral treatment; however, both wtVSV and VSV-S infection significantly enhanced NK cell cytotoxicity, as indicated by the increased population of CD107a^+^ NK cells (Fig. 6E and F). No difference in the number of cytotoxic NK cells was detected in MOC2 tumors between VSV-S and wtVSV infections (Fig. 6F). These observations indicate that VSV-S enhances CD8 + T cell tumor trafficking and cytotoxic activity more effectively than wtVSV.

### The combination of VSV-S and anti-PD1 monoclonal antibody (αPD1) enhances antitumor activity in the orthotopic syngeneic mouse model of head and neck cancer

**Fig. 7:**
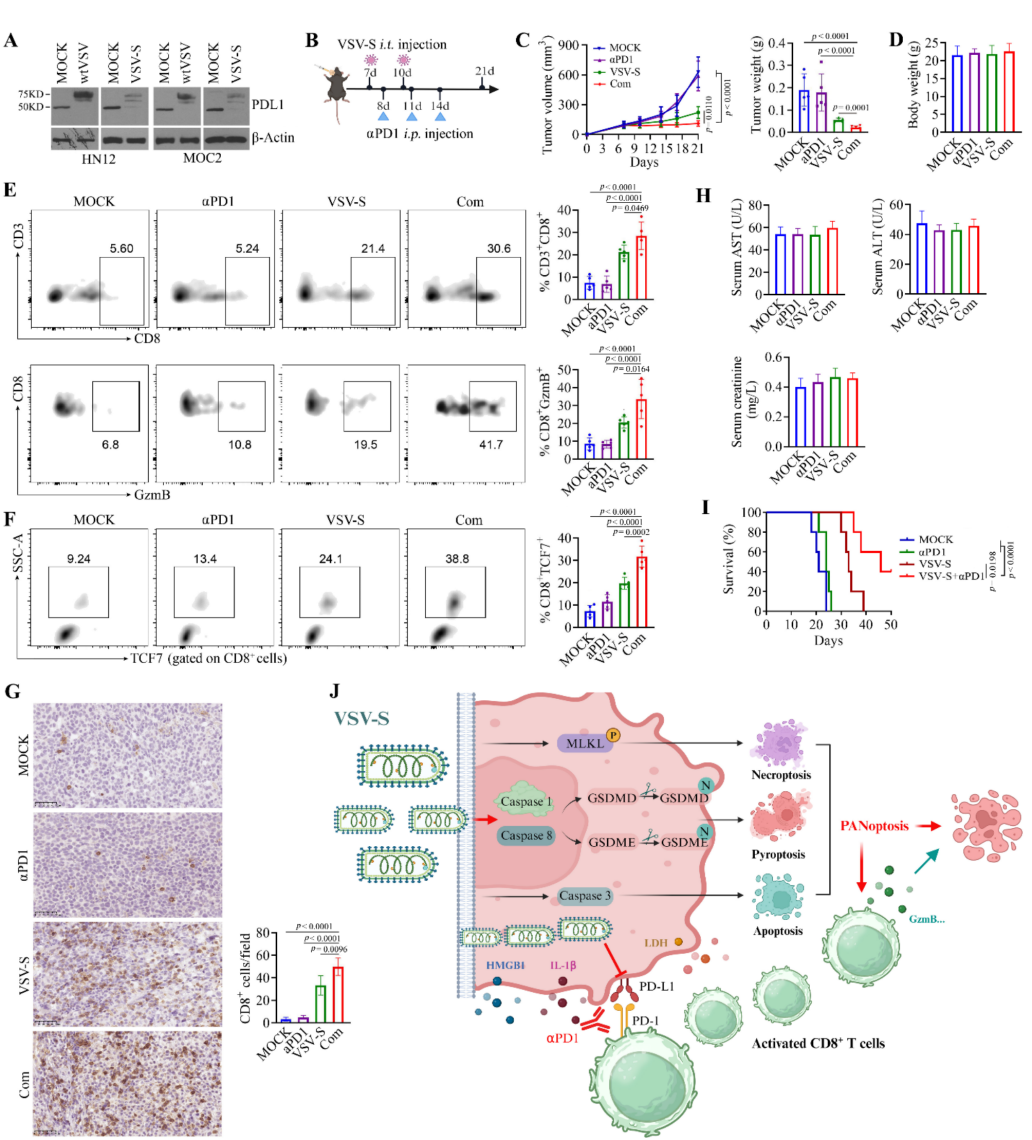
Addition of PD1 inhibition to VSV-S infection enhances antitumor activity in the orthotopic syngeneic mouse model of head and neck cancer. (**A**) Effect of wtVSV and VSV-S infection on PDL1 expression in both human HN12 cells and mouse MOC2 cells. (**B**) Schematic diagram of the treatment procedure for VSV-S infection alone or in combination with αPD1. (**C**) Growth curve and weight of MOC2 tumors isolated from C57BL/6 mice treated with VSV-S and αPD1, alone or in combination (*n* = 5 mice/group). (**D**) Body weight of MOC2 tumor-bearing mice treated with VSV-S and αPDL1, alone or in combination (*n* = 5 mice/group). (**E**) Percent of CD8^+^ T cells and cytotoxic (GzmB^+^) CD8^+^ T cell subsets in MOC2 tumors treated with VSV-S and αPD1, alone or in combination (*n* = 5 mice/group). (**F**) Percent of stem-like (TCF7^+^) CD8^+^ T subset in MOC2 tumors treated with VSV-S and αPD1, alone or in combination (*n* = 5 mice/group). In (**C**-**F**), statistical significance was evaluated using one-way ANOVA with Tukey’s adjustments for multiple comparisons. (**G**) IHC for CD8 in MOC2 tumor tissues isolated from C57BL/6 mice treated with VSV-S and αPD1, alone or in combination. Representative IHC images and quantitative data (*n* = 10 random field/slide/group) are shown in the left and right panels, respectively. Statistical significance was determined by unpaired, two-tailed Student’s t test. (**H**) Blood biochemical indexes (AST, ALT, and creatinine) of MOC2 tumor-bearing mice following the administration of VSV-S and αPD1, alone or in combination. (**I**) Survival curve of MOC2 tumor-bearing mice treated with VSV-S and αPD1, alone or in combination (*n* = 5 mice/group). Survival curves were compared using the Log-rank (Mantel-Cox) test for multiple groups. (**J**) A proposed model for VSV-S-mediated antitumor activity

Next, we sought to determine whether VSV-S affected PDL1 levels in HNSCC cells. Strikingly, both wtVSV and VSV-S infections nearly completely inhibited PDL1 expression in human HN12 cells and mouse MOC2 cells (Fig. 7A), suggesting that infection with these viruses may alleviate T cell exhaustion by suppressing the PDL1/PD1 signaling axis between tumor cells and T cells. We also assessed PDL1 levels in MOC2 tumors treated with MOCK, wtVSV, or VSV-S. Unlike in cell culture, PDL1 levels were significantly reduced following either wtVSV or VSV-S treatment; however, they were still detectable in tumor tissues (Supplementary Fig. S7). This may be due in part to the lower efficiency of in vivo viral infection compared to in vitro infection, providing a rationale for further combination of VSV-S with αPD1. We then treated MOC2 tumor-bearing C57BL/6 mice with VSV-S and/or αPD1 to evaluate their antitumor effects (Fig. 7B). Three weeks after cell inoculation, there was no difference in tumor growth curve and weight between the αPD1-treatment group and the control group (Fig. 7C). However, the addition of αPD1 to VSV-S infection significantly enhanced the antitumor activity of VSV-S (Fig. 7C), and this combination did not affect the body weight of tumor-bearing mice (Fig. 7D). This study also revealed that combining αPD1 with VSV-S resulted in greater antitumor efficacy compared to combining αPD1 with wtVSV in animal models (Supplementary Fig. S8), indicating that VSV-S enhances the synergy of PD1-based immunotherapy for improved antitumor effects.

The combination of VSV-S and αPD1 significantly increased both the number of tumor-infiltrating CD8^+^ T cells and their cytotoxicity compared to either treatment (Fig. 7E and F). Additionally, we observed an increase in stem-like (TCF7^+^) CD8^+^ T cells in MOC2 tumors that received the combination treatment compared to single-agent treatment (Fig. 7G). In cancer immunology, TCF7^+^ CD8^+^ T cells are thought to play a key role in promoting effective antitumor immunity by preserving a reservoir of memory T cells capable of rapidly responding to tumor antigens. The expansion of this T cell subset suggests that combining VSV-S with αPD1 may be more effective in developing immune memory and sustaining antitumor responses than either treatment alone.

Next, we measured serum levels of ALT, AST, and creatinine. There were no significant changes in AST, ALT, or creatinine levels among the tumor-bearing mice receiving VSV-S, αPD1, or their combination (Fig. 7H), suggesting that neither the single agent nor their combination caused hepatotoxicity or nephrotoxicity. Most notably, this combination treatment resulted in significantly longer survival in mice compared to either treatment alone (Fig. 7I). Collectively, VSV-S not only enhances CD8^+^ T cell-mediated antitumor immunity by inducing PANoptosis in HNSCC cells but also reverses T cell exhaustion by suppressing the expression of the key immune checkpoint regulator PDL1 in HNSCC cells (Fig. 7J). As the addition of αPD1 can further block the PD1/PDL1 signaling axis between tumor and immune cells, the combination of VSV-S and PD1-based immunotherapy deserves to be further investigated as a novel strategy for achieving more effective and durable antitumor effects.

## Discussion

OVs have recently been developed as an effective antitumor strategy by selectively infecting and destroying tumor cells. Lysis of tumor cells infected with OVs may release PAMPs and DAMPs and activate the innate immune system by interacting with pattern recognition receptors (PRRs) [28]. The detection of viral components by NK cells, macrophage cells and other surrounding immune cells can secrete inflammatory cytokines, such as IFNγ, IL-12, type I IFNs, TNFα, and IL-6, to trigger an immediate innate immune response [29]. On the other hand, the release of tumor-associated antigens (TAAs) during OV infection and their presentation by antigen-presenting cells (APCs), such as dendritic cells (DCs), primes and activates cytotoxic T cells. This initiates an adaptive immune response that specifically targets and kills tumor cells expressing TAAs [30]. Additionally, OVs can modulate the immune landscape by reducing the number of immunosuppressive cells within the TME, such as regulatory T cells (Tregs) and myeloid-derived suppressor cells (MDSCs) and increase the infiltration of cytotoxic immune cells [31]. Some OVs can also modulate the tumor vasculature, improving immune cell infiltration and function within the tumor [32]. Furthermore, OVs often lead to the generation of memory T cells that provide long-term surveillance against tumor recurrence [33]. These immune responses, both innate and adaptive, enhance the overall effectiveness of OVs against tumors by directly killing infected cancer cells and by establishing an environment that promotes continued immune-mediated tumor cell destruction. To generate a more effective oncolytic VSV, we have engineered VSV-S by inserting the SMAC gene after the M gene in the VSV genome [7]. Compared to wtVSV, VSV-S did not show a greater potential in activating NK cell-mediated innate immune responses. However, VSV-S significantly enhanced CD8^+^ T cell-mediated adaptive antitumor immunity, as evidenced by an increased number of tumor-infiltrating CD8^+^ T cells and their cytotoxicity compared to those observed following wtVSV infection. We also demonstrate that the therapeutic efficacy of VSV-S can be further enhanced when combined with αPD1. Future studies are warranted to determine whether VSV-S in combination with other treatments, such as radiation or targeted therapy, could yield additional benefits.

OVs are involved in the regulated cell death of tumor cells through several mechanisms. The first is the selective infection and replication within tumor cells, leading to tumor cell apoptosis. This is achieved through the activation of cellular pathways that lead to apoptosis, often triggered by viral proteins or the cellular stress response to viral infection. Specifically, OVs have the potential to induce intracellular redistribution of Ras, promoting apoptosis and the release of progeny viruses [34]. Moreover, OVs can induce other forms of regulated cell death, such as necroptosis and pyroptosis [35]. These processes can further enhance the antitumor immune response by releasing TAAs and inflammatory cytokines. In some cases, OVs trigger autophagy, a process where cells degrade their own components [36]. In another study, the inducer of ferroptosis was combined with OV to improve the efficacy of treatment [37]. Recent reports indicate that oncolytic VSV induces tumor cell pyroptosis by activating GSDME, resulting in an increase in cytotoxic T lymphocytes during VSV therapy [38]. Our study supports these findings and further demonstrates that the newly developed VSV-S not only enhances VSV-mediated pyroptosis but also induces PANoptosis to stimulate antitumor immunity. PANoptosis includes several characteristics of pyroptosis, apoptosis, and necroptosis, which plays a critical role in antitumor immune response [26]. Although the detailed mechanisms by which PANoptosis regulates antitumor immunity vary across different types of cancer, increasing evidence shows that key PANoptosis-related genes (PRGs), such as GSDM family proteins, NOD-like receptor protein 3 (NLRP3), caspase 8, receptor-interacting protein kinases (RIPK)3, and MLKL are highly associated with tumor progression and immune response [39, 40]. Here, we report that VSV-S induces PANoptosis more efficiently in HNSCC cells compared to wtVSV. Although there is optimism about the potential of new drugs that can induce PANoptosis for cancer treatment, PANoptosis-based therapies are still in their early stages. Our study introduces the novel approach of VSV-S to induce PANoptosis that could be a promising strategy when combined with ICIs.

Apoptosis, pyroptosis, and necroptosis follow distinct signaling pathways; however, the complexity of their associations indicates a dynamic network of molecular interactions rather than separate, isolated pathways [26]. Our study shows that both wtVSV and VSV-S trigger GSDME-dependent pyroptosis. Unlike wtVSV, VSV-S further enhances pyroptosis by activating the caspase 1/GSDMD signaling pathway, which is linked to increased ROS-related apoptosis. During VSV-S infection, inhibiting caspase 3-mediated apoptosis or reducing cellular ROS levels significantly downregulates caspase 1/GSDMD signaling, resulting in decreased cell pyroptosis in HNSCC cells. This suggests a crosstalk between VSV-S-induced pyroptosis and apoptosis. We showed that VSV-S induced increased HMGB1 secretion via pyroptosis in both human and murine HNSCC cells. HMGB1 is a nuclear protein that plays a crucial role in immune stimulation, particularly in the context of inflammation, infection, and cancer. Paradoxically, HMGB1 may also promote malignancy by enhancing tumor growth, metastasis, and immune evasion [41]. Therefore, despite the remarkable antitumor effects observed in mouse models following VSV-S treatment, the potential of VSV-S-induced pyroptosis may also act as a barrier to antitumor efficacy. Previous studies reported that GSDME-mediated pyroptosis promoted colitis-associated colorectal cancer by releasing HMGB1, which in turn induced tumor cell proliferation via ERK1/2 pathway [42]. To investigate this in the context of our study, we assessed ERK1/2 phosphorylation in HN12 and MOC2 cells with and without viral infection. Interestingly, we found that, in contrast to the reported findings, ERK1/2 phosphorylation was significantly downregulated in HNSCC cells infected with either wtVSV or VSV-S (Supplementary Fig. S9), suggesting that increased HMGB1 during viral infection cannot promote tumor growth via ERK1/2 activation. However, further studies are needed to elucidate the dual role of HMGB1 in VSV-S-based treatment, as it may act as both a pro-inflammatory mediator and a potential contributor to immune suppression.

Given that the majority of HNSCCs are located on or near the surface of the skin or mucous membranes (such as in the mouth, throat, or larynx), they are more accessible for direct injection. One potential advantage of administering VSV-S for HNSCC via intratumoral injection is the possibility of improving local tumor concentration while reducing systemic side effects. However, this delivery method may limit its clinical application compared to systemic administration, as local delivery might not effectively reach all tumor sites, especially in cases of metastatic disease, and could be logistically challenging for treating a large number of patients. Therefore, beyond evaluating therapeutic efficacy, it is crucial to investigate the safety and virus distribution in systemic administration of VSV-S in animal models. Achieving these goals will be a necessary step before advancing this approach to early phase human clinical trials.

## Conclusions

We provided evidence of how VSV-S enhances CD8^+^ T cell-mediated antitumor immunity and demonstrated that the combination of VSV-S with PD1 blockade offers a synergistic therapeutic strategy for HNSCC. These findings underscore the potential of this combination as an effective treatment approach for HNSCC patients.

## Electronic supplementary material

Below is the link to the electronic supplementary material.

Supplementary Material 1

## Data Availability

The datasets used and/or analyzed during the current study are available from the corresponding author on reasonable request.

## List of Abbreviations

ALT: Alanine Transaminase
ANOVA: Analysis of Variance
APCs: Antigen Presenting Cells
AST: Aspartate Transaminase
ATCC: American Type Culture Collection
BLI: Bioluminescence Imaging
CHX: Cycloheximide
DAMPs: Damage-associated Molecular Patterns
DCF: 2’,7’-Dichlorofluorescin
DCs: Dendritic Cells
DEPs: Differentially Expressed Proteins
DPBS: Dulbecco’s PBS
ELISA: Enzyme-linked Immunosorbent Assays
ER: Endoplasmic Reticulum
FBS: Fetal Bovine Serum
FFPE: Formalin-fixed Paraffin-Embedded
GSDMD-N: N-terminal Gasdermin D
GSDME-N: N-terminal Gasdermin E
HNSCC: Head and Neck Squamous Cell Carcinoma
IACUC: Institutional Animal Care and Use Committee
IHC: Immunohistochemistry
ICIs: Immune Checkpoint Inhibitors
IOD: Integrated Optical Density
LC-MS: Liquid Chromatography-Mass Spectrometry
LDH: Lactate Dehydrogenase
LNMets: Lymph Node Metastases
MDSCs: Myeloid-derived Suppressor Cells
MLKL: Mixed Lineage Kinase Domain-Like Protein
MOI: Multiplicity of Infection
NAC: N-Acetylcysteine
NLRP3: NOD-like Receptor Protein 3
OVs: Oncolytic Viruses
PAMPs: Pathogen-associated Molecular Patterns
PBMCs: Peripheral Blood Mononuclear Cells
PFU: Plaque-forming Units
PRGs: PANoptosis-related genes
PRRs: Pattern Recognition Receptors
RIPK: Receptor-interacting Protein Kinases
ROS: Reactive Oxygen Species
SD: Standard Deviation
shGFP: shRNA Targeting the Gene Encoding Green Fluorescent Protein
shGSDMD: shRNAs Targeting the Human *GSDMD* Gene
SI: Signal Index
SMAC: Second Mitochondria-Derived Activator of Caspases
TAAs: Tumor-associated Antigens
TCGA: The Cancer Genome Atlas
TEM: Transmission Electron Microscopy
TME: Tumor Microenvironment
Tregs: Regulatory T cells
VSV: Vesicular Stomatitis Virus
VSV-IFN-β: VSV Expressing Murine IFN-β
wtVSV: Wild-type VSV
