# Supplementary material for: SMAC-armed oncolytic virotherapy enhances the anticancer activity of PD1 blockade by modulating PANoptosis
Source: Biomark Res. 2025 Jan 9;13:8. doi: 10.1186/s40364-025-00726-w (PMC11721257; doi:10.1186/s40364-025-00726-w)

Supplemental Information for

SMAC-armed oncolytic virotherapy enhances the anticancer activity of PD1 blockade by modulating PANoptosis

Chen *et al*

Correspondence: Yong Teng, yong.teng@emory.edu

This PDF file includes:

Supplementary Table S1-Table S2

Supplementary Figure S1-Figure S9

Table S1. Antibodies used in this study

Resources	Identifier (Cat#)	Source (vendor)	Usage
**Primary antibodies**			
DFNA5/GSDME (EPR19859)	ab215191	Abcam	WB
GSDMD (EPR19829)	ab210070	Abcam	WB
IL-1 beta	ab254360	Abcam	WB
Pro Caspase-1 + p10 + p12 (EPR16883)	ab179515	Abcam	WB
Cleaved Gasdermin D (Asp276) (E3E3P)	#10137	Cell Signaling Technology	WB
Cleaved Caspase-3 (Asp175) (5A1E)	#9664	Cell Signaling Technology	WB
Caspase-1 (E2Z1C)	#24232	Cell Signaling Technology	WB
Cleaved Caspase-1 (Asp296) (E2G2I)	#89332	Cell Signaling Technology	WB/IHC
Cleaved Caspase-8 (Asp374) (18C8)	#9496	Cell Signaling Technology	WB
Phospho-MLKL (Ser345) (D6E3G)	#37333	Cell Signaling Technology	WB/IHC
Cleaved PARP (Asp214) (D6X6X)	#94885	Cell Signaling Technology	WB
CD8α (D8A8Y)	#85336	Cell Signaling Technology	IHC
CD8α (D4W2Z) XP	#98941	Cell Signaling Technology	IHC
CXCL9/MIG (E6Z5W)	#30327	Cell Signaling Technology	WB
CXCL10 (D5L5L)	#14969	Cell Signaling Technology	WB
PD-L1/CD274	17952-1-AP	Proteintech	WB/IHC
HMGB1	A25444	Abclonal	WB
Smac/Diablo	A8889	Abclonal	WB/IHC
HSP90	05-594-25UG	Sigma-Aldrich	WB
β-Actin (AC-74)	A5316	Sigma-Aldrich	WB
**Secondary antibodies**			
Goat Anti-rabbit IgG, HRP-linked Antibody	#7074	Cell Signaling Technology	WB
Goat Anti-mouse IgG, HRP-linked Antibody	#7076	Cell Signaling Technology	WB
**Flow cytometry**			
Anti-mouse CD45 (30-F11)	103116	Biolegend	Flow
Anti-mouse CD3 (17A2)	560527	BD Biosciences	Flow
Anti-mouse CD8a (53-6.7)	100722	Biolegend	Flow
Anti-human/mouse Granzyme B (GB1)	515406	Biolegend	Flow
Anti-mouse TCF-7/TCF-1 (S33-966)	564217	BD Biosciences	Flow
Anti-mouse NK-1.1 (PK136)	108740	Biolegend	Flow
Anti-mouse CD107a (1D4B)	553793	BD Biosciences	Flow

Table S2. Primers used in this study

Gene name	Forward primer (5’-3’)	Reverse primer (5’-3’)
CCL2	CCCCAGTCACCTGCTGTTAT	GGGTCAGCACAGATCTCCTT
CCL3	AGTTCTCTGCATCACTTGCTG	CGGCTTCGCTTGGTTAGGAA
CCL5	CAGTGGCAAGTGCTCCAACC	CCATCCTAGCTCATCTCCAAAGAGT
CXCL9	GTGGTGTTCTTTTCCTCTTGGG	ACAGCGACCCTTTCTCACTAC
CXCL10	GCAAGCCAATTTTGTCCACG	ACATTTCCTTGCTAACTGCTTTCAG
CXCL12	ATTCTCAACACTCCAAACTGTGC	ACTTTAGCTTCGGGTCAATGC
GAPDH	GAAGGTGAAGGTCGGAGTC	GAAGATGGTGATGGGATTTC

Figure S1. Representative phase contrast and fluorescent images of HN12 cells after infection with wtVSV for 4 hours. wtVSV expresses an mCherry-fused P protein that labels the infected cells with red fluorescence (right panel).

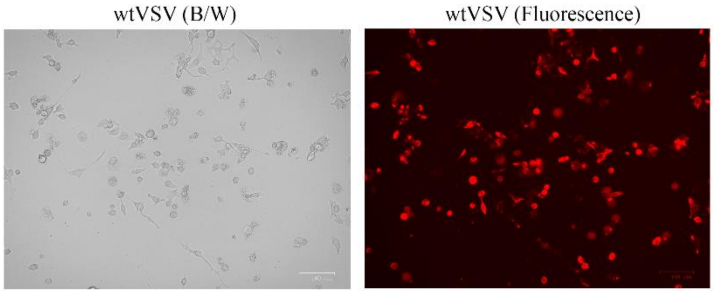

Figure S2. The quantitative Western blot data for the corresponding figures are designated as follows: Fig. 1C (A), Fig. 2A (B), Fig. 4A (C), Fig. 4C (D), Fig. 4F (E), and Fig. 4G (F). Data are presented as mean values +/- SD. Statistical significance was determined by unpaired, two-tailed Student's t test (n = 3 repeats).

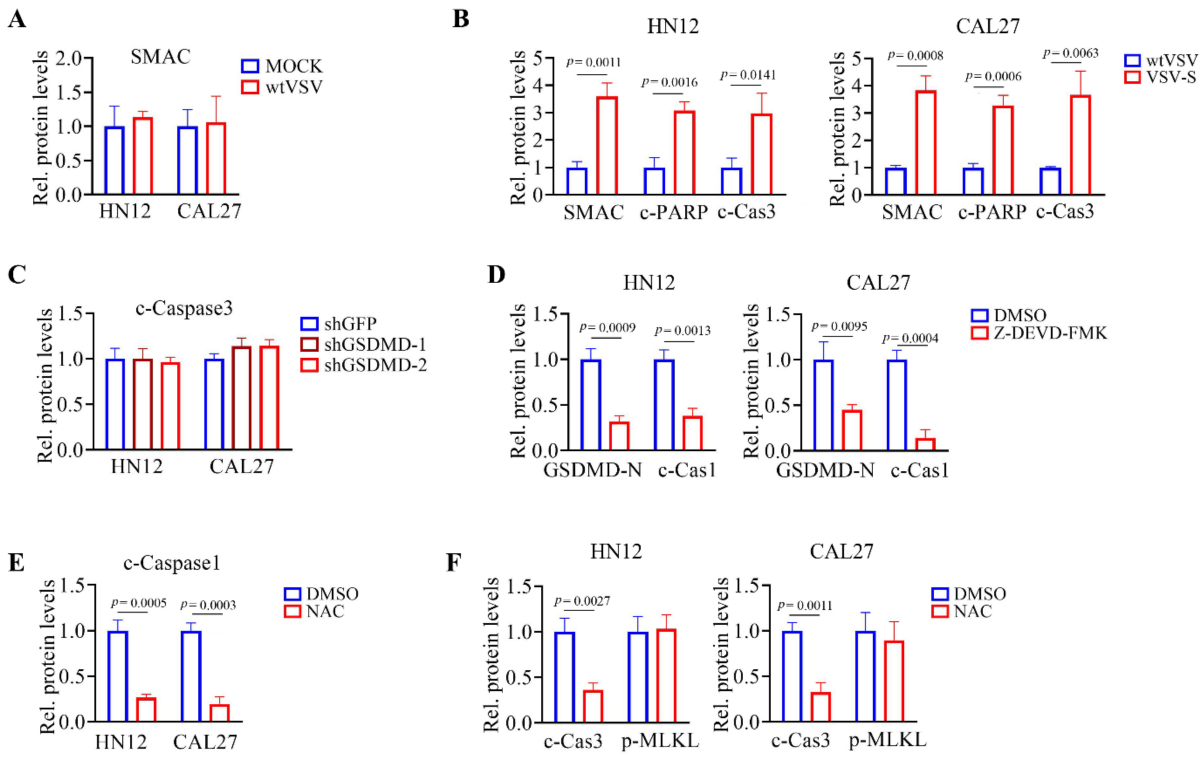

Figure S3. Representative flow images showing apoptosis of HN12 and CAL27 cells infected with wtVSV alone or in combination with AT-406.

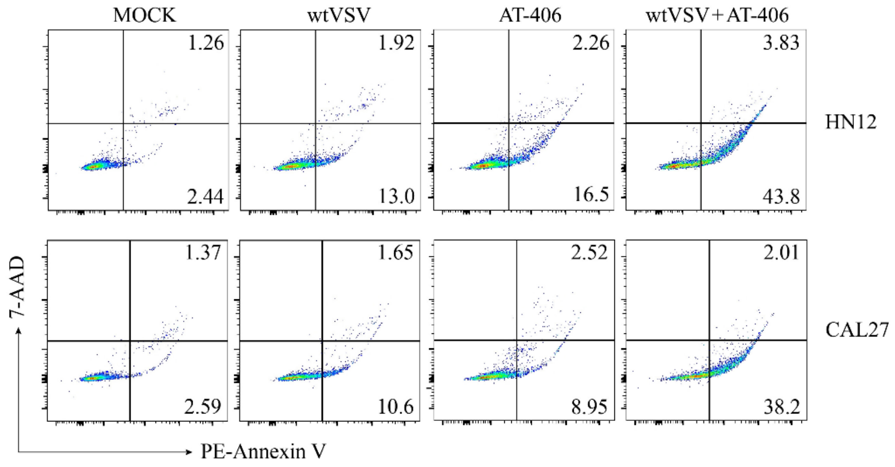

Figure S4. Changes in the expression and secretion levels of the indicated chemokines in HNSCC cells following wtVSV or VSV-S infection. The gene expression levels were assessed by qRT-PCR (A), while the protein secretion levels were analyzed by Western blot (B). Data are presented as mean values +/- SD. Statistical significance was determined by unpaired, two-tailed Student's t test (n = 3 repeats).

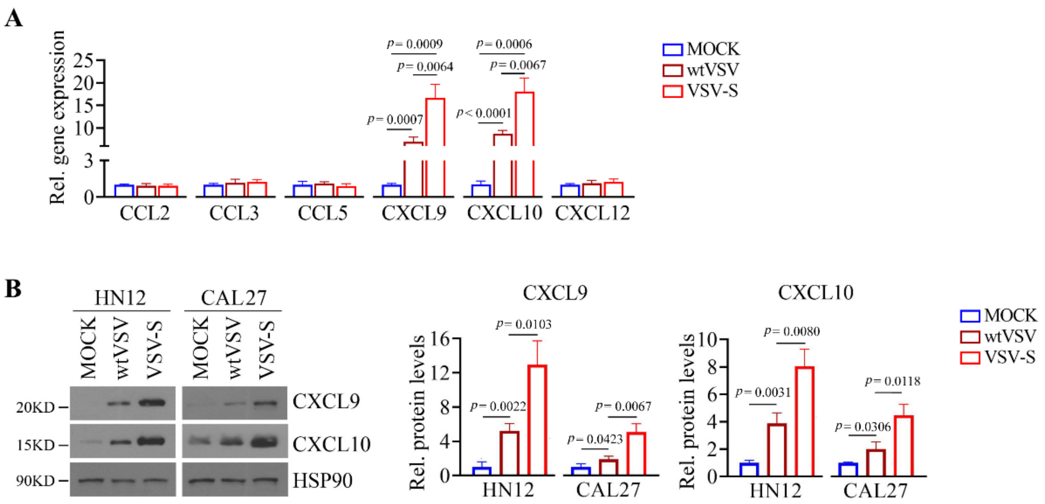

Figure S5. LC-MS analysis showing the top ten proteins with the highest sequence coverage uniquely identified in the supernatants of MOC2-ER cells after VSV-S infection (*vs.* MOCK).

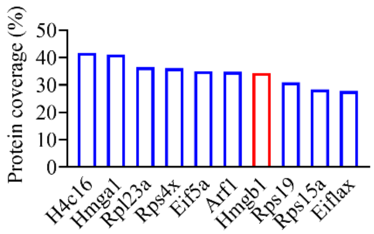

Figure S6. Representative spectrograms showing four tryptic peptides derived from HMGB1 protein identified by LC-MS analysis.

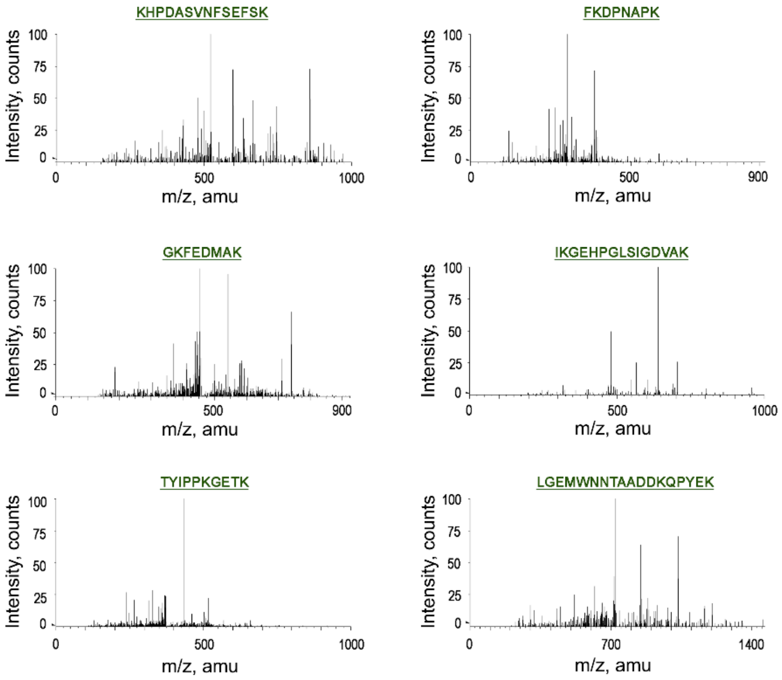

Figure S7. IHC for PDL1 in MOC2 tumor tissues isolated from C57BL/6 mice treated with MOCK, wtVSV or VSV-S. Representative IHC images and quantitative data (n = 10 random field/slide/group) are shown in the left and right panels, respectively. Data are presented as mean values +/- SD. Statistical significance was determined by unpaired, two-tailed Student's t test.

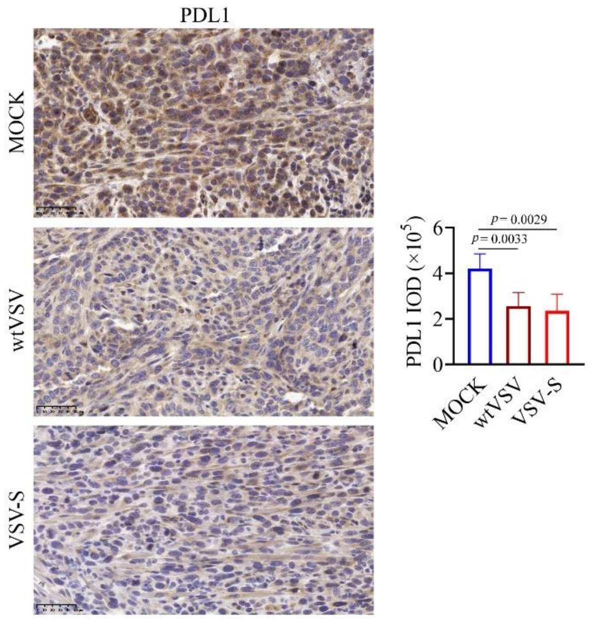

Figure S8. Growth curve (A) and weight (B) of MOC2 tumors isolated from C57BL/6 mice treated with MOCK, the combination of wtVSV and αPD1, or the combination of VSV-S and αPD1. On day 7 after MOC2 cell inoculation, a dose of 3×10^6 PFU of wtVSV or VSV-S was administered by intratumoral injection every three days for a total of two doses. InVivoMAb anti-mouse PD1 at the dose of 200μg per injection was administered intraperitoneally every three days for a total of three doses, starting one day after the first dose of wtVSV or VSV-S. Data are presented as mean values +/- SD. Statistical significance was evaluated using one-way ANOVA with Tukey's adjustments for multiple comparisons (n = 5 mice/group).

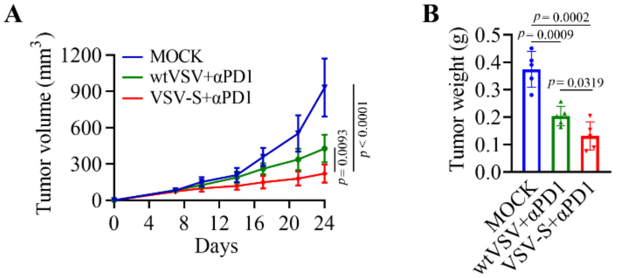

Figure S9. Changes in the levels of total and phosphorylated ERK1/2 following wtVSV or VSV-S infection analyzed by Western blot. Data are presented as mean values +/- SD. Statistical significance was determined by unpaired, two-tailed Student's t test (n = 3 repeats).

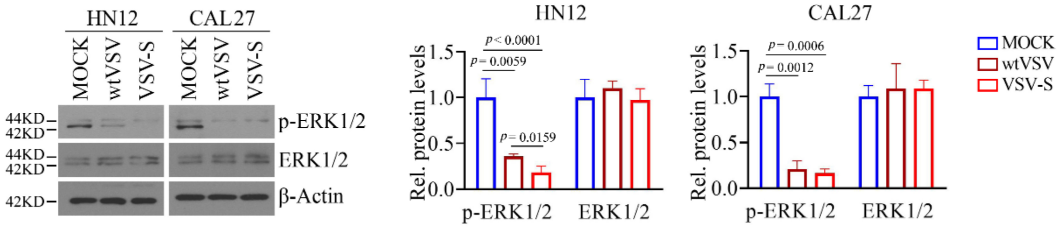

Supplement: Supplementary file 1 — Supplementary Material 1 [file 40364_2025_726_MOESM1_ESM.pdf]
